# Targeting Antitumor Immune Response for Enhancing the Efficacy of Photodynamic Therapy of Cancer: Recent Advances and Future Perspectives

**DOI:** 10.1155/2016/5274084

**Published:** 2016-09-08

**Authors:** Yamin Yang, Yue Hu, Hongjun Wang

**Affiliations:** ^1^Department of Biomedical Engineering, Nanjing University of Aeronautics and Astronautics, 169 Sheng Tai West Road, Nanjing, Jiangsu 211106, China; ^2^Department of Biological and Environmental Engineering, Cornell University, 120 Riley Robb, Ithaca, NY 14853, USA; ^3^Department of Chemistry, Chemical Biology and Biomedical Engineering, Stevens Institute of Technology, Castle Point on Hudson, Hoboken, NJ 07030, USA

## Abstract

Photodynamic therapy (PDT) is a minimally invasive therapeutic strategy for cancer treatment, which can destroy local tumor cells and induce systemic antitumor immune response, whereas, focusing on improving direct cytotoxicity to tumor cells treated by PDT, there is growing interest in developing approaches to further explore the immune stimulatory properties of PDT. In this review we summarize the current knowledge of the innate and adaptive immune responses induced by PDT against tumors, providing evidence showing PDT facilitated-antitumor immunity. Various immunotherapeutic approaches on different cells are reviewed for their effectiveness in improving the treatment efficiency in concert with PDT. Future perspectives are discussed for further enhancing PDT efficiency via intracellular targetable drug delivery as well as optimized experimental model development associated with the study of antitumor immune response.

## 1. Introduction

**Figure 1:**
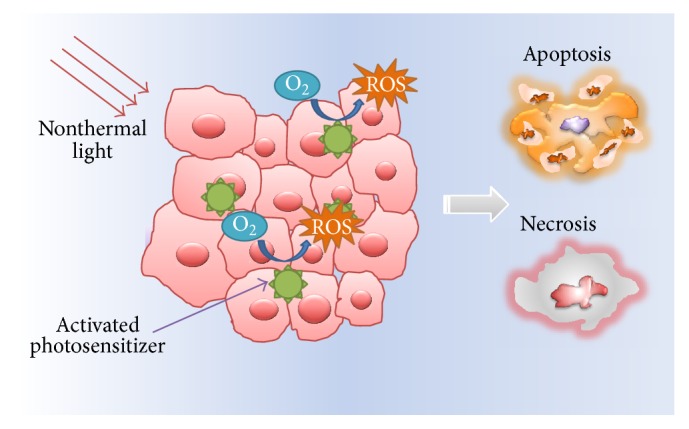
The mechanisms of PDT that can induce apoptosis and necrosis of tumor cells.

Photodynamic therapy (PDT) has been developed as a minimally invasive therapeutic modality for the treatment of various cancers as well as nonmalignant diseases [1, 2]. PDT is formulated based on the “photodynamic” reaction of photosensitizer upon light irradiation. Three major components are involved in PDT process: nontoxic photosensitizers, harmless light, and tissue oxygen [3–5]. As shown in Figure 1, photosensitizers are molecules that can be excited by the light of a particular wavelength. Activated photosensitizers under light irradiation then transfer energy to neighboring molecular oxygen, culminating in the generation of highly reactive oxygen species (ROS). Consequently, those unstable ROS can destroy targeted cells via apoptotic or necrotic process or the combination. Owing to the selective uptake of photosensitizers by tumor cells, confined light exposure to specific area, minimally invasiveness feature, and relative simple procedure, PDT shows its intrinsic advantages including tumor targeting capability, low morbidity, and good patient tolerance [6–8].

The* in vivo* microenvironment of solid tumor can be essentially classified into (1) the bulk of solid tumor made up by tumor cells; (2) vasculature surrounding the tumor structure formed by endothelial cells; and (3) interstitial space comprised of stromal cells and immune cells [9, 10]. It is well acknowledged that PDT-mediated tumor destruction* in vivo* involves three distinct mechanisms targeting all above three major components of solid tumor tissue: (1) PDT-generated oxidative stress can directly cause apoptosis and necrosis of tumor cells; (2) PDT can destruct tumor-associated vasculature, leading to tumor infarction; (3) PDT can activate an acute inflammatory and induce host-defense immune response that can consequently kill tumor cells [11, 12].

Despite its multifaceted advantages, PDT has not yet become the mainstream of cancer intervention mainly due to its insufficient therapeutic efficacy, low targeting capability of currently available photosensitizers, and limited light penetration for deep tumor tissues [13, 14]. In an effort to enhance the effectiveness of anticancer PDT, many studies have been conducted to develop photosensitizers with improved photosensitivity and high quantum yield of singlet oxygen, as well as efficient delivery of photosensitizers at therapeutic concentrations to target cells. Novel strategies in PDT have been developed over the past several decades, including the development of nanomaterial-based platforms for selective photosensitizer delivery or light source and optic catheters for increased tissue penetration at longer wavelengths [15, 16], whereas, focusing on improving direct cytotoxicity to tumor cells through various techniques, vascular-targeted photodynamic therapy takes advantage of intravascular excitation of a photosensitizer to produce cytotoxic ROS that could induce vascular damage including local thrombus formation, vascular occlusion, and tissue hypoxia [17, 18]. The microvascular stasis and resulting hypoxia in tumor areas in PDT are effective means to produce cytotoxicity and tumor regression. Tumor vasculature-targeting PDT can be achieved by effectively manipulating the photosensitizer drug administration and light illumination interval (drug-light interval) during PDT treatment or by the modification of photosensitizer molecular structure. For instance, shorter photosensitizer-light intervals could be applied for tumor vasculature targeting by confining photosensitizer localization within blood vessels. Tumor endothelial markers could be modified on photosensitizers to achieve active vascular targeting of PDT [19].

Besides above techniques, recognizing the importance of the immunological contribution to the effect of PDT, there are growing interests in developing approaches to induce and facilitate the antitumor immune response following PDT. In this review we will discuss the effect of PDT of cancer on the immune response, summarize recent progress on employing PDT-induced antitumor immunity to achieve better therapeutic outcomes, and propose future perspectives for better design of PDT protocols based on the understanding of tumor-immune system interaction.

## 2. PDT-Induced Immune Response

**Figure 2:**
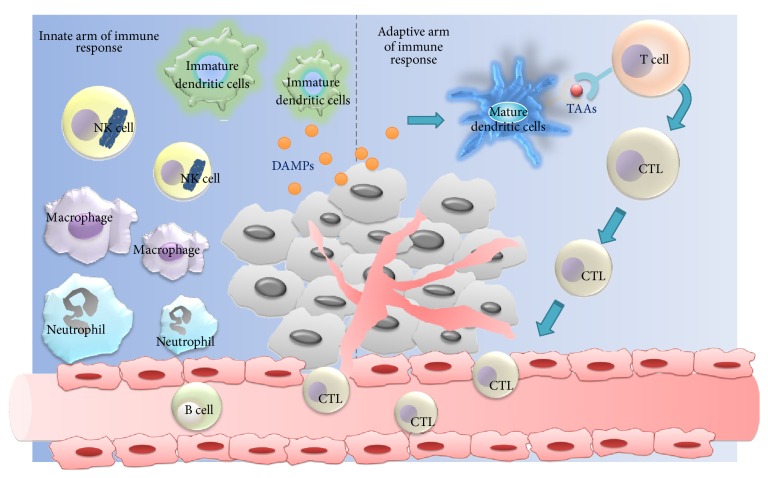
PDT-induced innate and adaptive arm of immune response.

Although PDT is a local treatment, tumor structure destruction is accompanied by acute inflammatory response with infiltration of leukocytes into tumor site and production of proinflammatory factors and cytokines [20]. Meanwhile, damaged/dying tumor cells will invoke a systemic antitumor immune response, which thereby mediated a secondary cause of tumor cell death. As shown in Figure 2, PDT is able to effectively stimulate both the innate and the adaptive arm of the immune system by exposing PDT-treated tumor cells to corresponding immune cells [21–24].

### 2.1. PDT-Induced Innate Arm of Immune Response

The innate arm of immune system reacts to pathogenic invaders by phagocytes (macrophages, neutrophils, and dendritic cells, DCs), the complement cascade, and natural killer (NK) cells. Followed by an acute inflammatory response, PDT-induced activation of the innate immune system consists of cytokine release, complement activation, recruitment, and activation of innate immune cells [25, 26]. After treatment, PDT-triggered oxidative stress results in extended tumor tissue injury. Damaged and/or dying tumor cells would present damage-associated molecular patterns (DAMPs) on their surface or release DAMPs into extracellular matrix. Those DAMPs act as danger signals and can be recognized and neutralized by above innate immune phagocytes, and then tumor cellular debris is eliminated [27]. Therefore, inducement of immunogenic DAMPs expression after PDT could be a potent way for attacking tumor cells by immune system based on a mechanism similar to fight against microbial invasion.

### 2.2. PDT-Induced Adaptive Arm of Immune Response

The adaptive arm of immune system involves antigen presenting cells (APC) that can trigger naive T cells to become cytotoxic tumor-specific T lymphocytes (CTLs) and antigen that can be recognized by B cells for eventual antibody production. PDT-induced immunogenic apoptosis of cancer cells (i.e., immunogenic cell death, ICD) trigger an effective antitumor immune response through activation of DCs and consequent activation of specific T cell response [28]. DCs are the most potent APC that link innate and adaptive immune response. Upon recognition of DAMPs exposed and/or released by damaged/dying tumor cells after PDT, DCs can transit to mature state and migrate to lymph nodes, present tumor-associated antigens (TAAs) to naive T cells, resulting in the generation of CTLs that attack and remove residual cancer cells. The mature DCs express peptide-major histocompatibility (MHC) complexes at the cell surface, prime CD4+ T helper cells and CD8+ to CTLs, and initiate an adaptive immune response [23, 29, 30].

## 3. Strategies for Enhancing Efficacy of PDT: Targeting Antitumor Immune Response

As discussed above, in addition to direct effects on tumor cells through generating either apoptosis and/or necrosis and PDT-triggered vascular damage that lead to tumor destruction, PDT owns its unique features in stimulating the host immune system. PDT-induced immune response not only enhances the efficacy of PDT by systemically eliminating residual tumor lesions and thus controls primary tumor growth, but also owns it particular promise in fighting for cancer cells in metastatic stage and thus inhibiting secondary disease [31]. Therefore, it is of great significance in the development of enhancing the antitumor immunity stimulated by PDT to achieve therapeutic efficacy. However, in most cases, PDT alone is insufficient in causing immune response that would lead to complete tumor killing. It is because, by dying through nonimmunogenic pathways, secreting immunosuppressive cytokines or other protumorigenic molecules, tumor cells could nurture a microenvironment that inhibits anticancer immunity. Therefore, to fully explore the exclusive immunogenic features in the microenvironment during PDT and to achieve effective immune response that can destroy tumor cells, it often requires the engagement of the synergistic effects of immunotherapy together with PDT [24].

### 3.1. Introducing of Immunostimulators in PDT-Induced Immune Response

In order to elicit an immune response that is strong enough to achieve superior tumor cures, one of modalities is to introduce immunostimulators in combination with PDT. In a previous review, St Denis et al. summarized investigations about immunostimulant therapies combined with PDT to potentiate the already robust immune response by PDT and further enhance its capability to attack malignant tumors [32]. The immunostimulatory agents combined with PDT can be mainly divided into microbial vaccines, cytokines in inflammatory cascades such as complement activators, growth factors, hormones, and other exogenous immunoadjuvants such as glycated chitosan [33]. The liberated tumor antigens after PDT together with the copresence of these immunostimulatory agents resulted in a significantly enhanced antitumor immune response with a better therapeutic outcome. Xia's group employed CpG oligodeoxynucleotide as immunoadjuvant to combine with PDT for the treatment of 4T1 metastatic breast cancer in a BALB/c immunocompetent mouse model. As CpG can prime immature DCs towards maturation and activation, they reported that peritumoral injected CpG improved local tumor control and showed a survival advantage in mice after PDT [34].

When combining PDT with immunostimulant therapies, the design of applied immunostimulators with effective delivery dosage definitely worth careful consideration. Another critical issue regarding this approach is the administration route for delivering those immunostimulatory agents to targeted tumor areas. In general, additional immunostimulators involved in PDT-induced immune response can be introduced through traditional intravenous or intratumoral injection. However, systemic delivery through intravenous route may result in high concentrations of immunostimulating drugs through blood circulation. As a result, powerful activation of immune system could be stimulated and eventually boost severe systemic immune response that is very harmful or even fatal [32]. Therefore, local delivery of immunotherapies through intratumoral administration is considered as better method to restrict those immunoadjuvants with desirable concentration at confined tumor area, while lowering the incidence and the intensity of their side effects. However, besides its invasive nature, intratumoral injection is only suitable for those readily accessible tumors of sufficient size [35, 36]. This practical limitation could be a problem especially if repeated injections are needed to trigger the adaptive immune response or when the tumors already undergo the metastasis stage in the blood. To address above issues regarding intravenous or intratumoral injection, the combination of well-designed drug delivery system holds significant potentials. For example, drug coated microneedles with minimal invasive feature have been developed to effectively deliver drugs to localized cancers in intratumoral injection [37]. Versatile drug delivery device with tumor targeting capability has been used for specific intratumoral homing of immunostimulating drugs through systemic circulation [38]. Taking advantage of stimulus-responsive drug delivery device, controlled release of immunostimulators in locoregional tumoral area after systemic administration could be activated externally. For instance, release behavior of immunostimulators encapsulated by photoactivated nanoparticles can be controlled by changing the light wavelength in the focused tumor area.

### 3.2. Epigenetic Modification to Enhance Tumor-Associated Antigen (TAA) Expression

Development of systemic adaptive immune response after PDT involves the maturation of DCs, the presentation of TAAs by DCs, and the priming of CTL response. The ultimate therapeutic outcome strongly depends on the efficacy of antigen presentation and recognition of tumor antigens by immune cells. Various approaches have been examined to accelerate the priming cells towards expression of high level of TAAs. One of the approaches to increase immunogenicity of targeted tumor cells focused on their genetic modification. Wachowska et al. have shown that PDT can induce strong antigen-specific antitumor immunity towards tumors expressing P1A antigen, which is a type of TAA. They have further combined PDT with a clinically approved epigenetic agent 5-aza-20-deoxycytidine that can induce expression of P1A antigen in different cancer cells. From their results, following high expression level of P1A antigen after epigenetic modification, potentiated antitumor effects and long-term survival after PDT have been found in tumor-bearing mice as a result of strong specific antitumor immune responses [39–41].

### 3.3. Investigation of PDT-Induced Antitumor Immunity on Different Cells

After the initial PDT-induced destruction of tumor structure, there will be either the direct interaction between immune cells and tumor cells or the influx and activation of immune cells caused by local inflammatory reaction. While the main focus of PDT is tumor cells, targeting immune cells by boosting their antitumor immunity or eliminating their role in processes such as immunosuppression could be an additional way to further increase the treatment effectiveness.

#### 3.3.1. PDT-Induced DAMPs Expression on Tumor Cells

DMAP expression in tumor cells plays significant role in activating DCs towards phenotypic and functional maturation. Evidences have shown that the DAMPs exposure follows PDT-induced ICD. Best characterized DAMPs induced by PDT include heat shock proteins 70 (HSP70) [42–45], calreticulin (CRT) [46], and high mobility group box 1 (HMGB1) [47]. It has also been found that the expression of DAMPs is associated with the type of photosensitizer. So far, photosensitizers that are able to trigger surface exposure of typical DAMPs on cancer cells include photofrin [27], hypericin [48], foscan, 5-aminolevulinic acid (5-ALA) [49], and Rose Bengal acetate [50]. However, while many of these PDT regimens have been shown to induce various DAMPs, DAMPs expression on tumor cells induced by PDT alone seldom reached all the necessary molecular determinants of ICD towards total tumor depletion. Therefore, it often requires the involvement of external resources or other simulators that can provide or generate additional DAMPs. For example, CRT is recognized as one of the pivotal DAMPs exposed on the surface of tumor cells treated by PDT. Korbelik et al. added recombinant CRT externally in SCCVII tumor-bearing immunocompetent C3H/HeN mice following PDT treatment. Their results revealed that recombinant CRT added before photosensitizer injection could boost enhanced antitumor response and produced improved PDT effect as compared to the group treated with PDT alone [51].

#### 3.3.2. Dendritic Cells

DCs play significant role in priming naive T cells to CTLs, which link innate and adaptive immune response. The activation of DCs followed by phenotypic and functional maturation is very crucial for TAA presentation and CTLs formation [52]. Cheong et al. manipulated the generation of intracellular ROS by the use of photosensitizer hematoporphyrin (HP) and examined their specific role in activating DCs. They found their transient photogeneration of intracellular ROS can efficiently induce DC maturation and potently enhance its migration* in vitro* and* in vivo* without significant cellular death. This strategy that can cause immature DCs towards effective vaccine adjuvants through photodynamic reaction is sufficient to initiate adaptive T cell responses* in vivo* [53].

Recent cell-based therapeutic strategy by introducing external DCs after PDT demonstrated an improvement in the outcome of PDT. Saji et al. have reported that tumor destruction with combination therapy of intratumoral injected DCs and ATXS10Na(II)-PDT against CT26 tumors in BALB/c mice was greater than that of individual therapies [54]. Jalili's group found that local PDT followed by intratumoral injection of naive DCs can prime cytotoxic T cells, which significantly inhibited the growth of subsequent tumors and led to tumor regression at distant sites, including multiple lung metastases [55]. These results not only confirmed the critical functions of DCs in regulating the PDT-induced immunity, but also suggested that injection of DCs could be a feasible method to invoke sufficient immune response and promote tumor cell killing in PDT.

#### 3.3.3. Macrophages

Monocytes can differentiate into two different macrophage phenotypes, M1 and M2. Whereas M1 macrophages are mostly involved in immune activation and invader attack, M2 macrophages play a role in promoting tumor progression and damage healing. M2 macrophages that function in suppressing antitumor immune response are termed as tumor-associated macrophage (TAM), which is actually a valid target for antitumor therapy [56–58]. There are therapeutic approaches exploiting the role of TAMs in PDT response. Hamblin's group has conducted series of studies regarding selective destruction of resident tumor-promoting TAMs under PDT. Attaching photosensitizers to ligands of the scavenger receptor can selectively destruct TAM and optimize PDT outcomes. They have demonstrated that maleylated albumin conjugated with photosensitizer chlorin (e6) can effectively target TAMs residing within the tumor through intratumoral injection, which is responsible for enhanced antitumor immune response and highly selective PDT killing [59, 60].

M2 macrophage-targeted photosensitizer delivery can be facilitated by the nanoparticles technology. Wen et al. utilized natural noninfectious nanoparticle cowpea mosaic virus (CPMV)/dendron hybrids as vehicle to specifically deliver Zn-EpPor photosensitizer to M2 macrophages as well as cancer cells. They observed differences in uptake of photosensitizer between the M1 and M2 populations and tested its efficiency in PDT of B16F10 melanoma cells [61]. It is implied that using a nanocarrier would be advantageous for tuning the delivery of photosensitizer to particular subpopulation of macrophages and would likely achieve either enhancement of immunostimulatory or elimination of immunosuppressive effects.

#### 3.3.4. T Lymphocytes

T lymphocyte activation during cancer immunotherapy, especially cytotoxic CD8+ T effector cells, plays an important role in killing tumor cells. PDT-induced cancer cell death causes acute inflammation, which might augment immunity by forming more effective cytotoxic T lymphocytes [62]. Gao et al. have found that PDT via phthalocyanine dye-labeled probe (DSAB-HK) not only significantly inhibited the growth of subcutaneous 4T1 tumors, but also subsequently triggered maturation of dendritic cells, resulting in the recruitment of CD8+ cytotoxic T lymphocytes for immune response. In addition, they employed monoclonal antibodies programmed death-1 (PD-1) to block inhibitory receptors on tumor cells known to evade host immune response through a process of T lymphocyte exhaustion. As a result, the synergistic effect of PDT and immune checkpoint inhibition could facilitate primary tumor eradication as well as eliminating the metastatic lesions [63].

Recognizing their crucial function in PDT-induced immunity, in addition to simulate DCs maturation and prime T lymphocytes, another alternative is to increase the number of T lymphocytes involved in PDT process. Blaudszun et al. reported their proof-of-concept study showing an innovative combination of adoptive transferred T lymphocyte mediated drug delivery, cellular immunotherapy, and PDT.* Ex vivo* activated human donor T cells were loaded with photosensitizer mTHPP and used as targeted delivery system for both adoptive T cell therapy and PDT. Bispecific antibody-redirected T lymphocytes possess dual specificity for recognizing both surface marker, such as TAA on the target cell, and a component of CD3 on T lymphocytes. When cocultivated with EpCAM-expressing human carcinoma cells, T lymphocytes induced the cytolytic activity, and at the same time photosensitizer was transferred to target cells. Consequently, significant improved cytotoxic capacity of photosensitizer boosted T cells was found after irradiation [64].

Besides cytotoxic T lymphocytes, tumor microenvironment may also induce immunosuppressive cytokine production and promote the proliferation of regulatory T cells (Tregs). Tregs can inhibit the process through which immature T cells became cytotoxic T cells, and unrestrained expansion of Treg may foster cancer progression [65]. Therefore, the depletion of Treg* in vivo* is considered to offer a rationale to facilitate tumor eradication and enhances antitumor immunity [66]. Experimental results showed that interleukin- (IL-) 6 was significantly increased in cells treated after hypericin-PDT [67]. And due to increased IL-6 levels, PDT downregulates the function of Treg in patients with invasive esophageal squamous cell carcinoma (ESCC) [68]. Reginato et al. found that the administration of cyclophosphamide CY before PDT led to absolute decreased numbers of Treg and the depletion of Treg potentiated PDT-mediated immunity, leading to complete tumor regression and long-term survival [69].

## 4. Future Perspectives of Improving Anticancer Immune Response after PDT

### 4.1. Intracellular Delivery of Photosensitizer

PDT-triggered cell death is closely associated with the intracellular localization and binding sites of photosensitizer [70]. Photoactivation of a mitochondrion-localized photosensitizer causes the release of cytochrome c, which is responsible for activation of apoptosis caspases. Besides mitochondria, damage to endoplasmic reticulum (ER) by PDT causes the release of Ca^2+^, which can also promote apoptosis [71, 72]. PDT can be used to spawn subcellular organelle-specific stress, and to our interest, subcellular organelle-dependent oxidative stress is closely related to signaling pathways in immunogenic cell death [73, 74]. Therefore, by manipulating the distinctive subcellular localization of photosensitizer, we may control both PDT process and antitumor immune response in tumor cells towards improved therapeutic outcomes.

#### 4.1.1. Endoplasmic Reticulum- (ER-) Targeting PDT

ER is at the center of a number of vital cellular processes, and ER functions have implications for various pathologies including cancer. ER stress is a key player of intracellular signaling pathways that govern ICD [75, 76]. Most of the agents inducing ICD are targeting ER. In a recent review, Garg et al. comprehensively discuss possible strategies enabling ER stress for cancer therapy [77].

It has been known that photosensitive drugs such as hypericin or mTHPC are predominantly localized in ER and can cause massive production of ROS-based ER stress upon light irradiation [78]. Such ER-localized photosensitizers not only will initiate ROS-triggered cell damage but also have the high probability of inducing ICD [79, 80]. Therefore, ER is of great interest in promoting PDT efficiency through both direct effects on tumor cells and indirect effects on immune system.

However, one critical issue that will possibly limit the applications of these photosensitizers remains to be their intrinsic absorbance wavelength of which light cannot penetrate into deep tissue. As a consequence,* in vivo* treatment with PDT via hypericin (~590 nm) or mTHPC (~415 nm) can only be performed on superficial neoplasm tissue. To fully explore their capability in treating deep tumor tissue, it would be necessary to retain their ER localization and expand their light absorption wavelength to the near-infrared region through modification with derivatives.

#### 4.1.2. Mitochondria-Targeting PDT

As the major cellular component where the photodynamic reaction occurs, localization of photosensitizers in mitochondria would provide more efficient cell killing in PDT. Mitochondria have been proposed to be the most effective subcellular targets for PDT [81, 82]. In our previous study, mitochondria-targeting gold nanoparticles were conjugated with triphenylphosphonium (TPP) molecules and used as vehicles to deliver photosensitizer precursor 5-ALA to achieve significantly elevated ROS formation and improved selective destruction of breast cancer cells in PDT [83]. Xu et al. fabricated a dual-targeting nanosystem and incorporated cationic porphyrin derivative (MitoTPP) with the polyethylene glycol- (PEG-) functionalized and folic acid-modified nanographene oxide (NGO) that can target cancer cells overexpressed with folate receptor and subsequently accumulate in mitochondria. Upon light irradiation, the released MitoTPP molecules generate singlet oxygen and cause enhanced oxidant damage to cells [84]. Soler et al. have shown that silicon phthalocyanine Pc 4-PDT located in mitochondria exerts an enhanced apoptotic effect on activated CD3+ T cells that may be exploited in targeting T cell-mediated skin diseases [85].

In addition to increase of ROS production, more interestingly, photodynamically induced mitochondrial apoptosis induces signal transduction pathways, which also participate in the development of immune responses [86]. Marrache et al. reported their study regarding the use of mitochondria-targeted-nanoparticle delivery systems to deliver mitochondria-acting photosensitizer for generation of tumor cell antigens to activate DCs* ex vivo* for potential immune response. They have found that mitochondria-targeted delivery of photosensitizer yielded significant high level of IL-18 secretion of breast cancer cells upon light stimulation, which contribute to remarkable production of interferon-gamma (IFN-*γ*) of activated DCs. Therefore, it suggests that targeting mitochondria for photosensitizer delivery could be a unique approach to generate effective tumor cell antigens and amplify the host immune responses following PDT [87].

### 4.2. The Involvement of Nanotechnology

Nanomaterials refer to the materials that have at least one dimension less than 100 nm, which thus provide superior characteristics [88]. In reorganization of current challenges of PDT, nanotechnology has been emerging to improve the potency of PDT efficacy. Nanomaterials can be utilized as passive carriers of photosensitizers [89, 90] to increase therapeutic concentration accumulated in tumor area while minimizing the collateral toxicity to nonmalignant cells. They can also serve as active participants [91, 92] in photodynamic reaction to further enhance the treatment effectiveness [93–96]. In addition to their direct action on tumor cells particularly their targeting capability on tumor tissues via intriguing Enhanced Permeability and Retention (EPR) effect [97–99], the unique effects of nanomaterials on immune system have also attracted growing attention recently in PDT.

Nanomaterials also represent an innovative strategy for delivering multiple immunostimulatory agents such as external antigens in a controllable and targeted manner. Or nanomaterial-based system can serve as inducers of apoptosis for release of internal antigens or inducers of cytokine production [100–102]. Aldinucci et al. have demonstrated that DCs differentiation and activation can be modulated when interfaced with carbon nanotubes at certain immunogenic profile [103]. Xiang et al. utilized antigen-loaded upconversion nanoparticles (UCNPs) to label and induce DC maturation and cytokine release. The nanoparticle-pulsed DC vaccine could be a promising strategy for DC-based immunotherapy against cancer. Moreover, UCNPs themselves in particular can be utilized as photosensitizer under irradiation with near-infrared light [104].

Of particular interest, owing to their controllable size and large surface area allowing for multiple functionalization with various ligands, nanomaterials are ideal carrier of photosensitizers and immunostimulatory agents, while providing intracellular targeting capabilities. For instance, nanocarriers (polymeric nanoparticles, liposomes, etc.) consisting of photosensitizers and antigens can be further modified with a mitochondria-targeting moiety (TPP or MTS) and thus guide both therapeutic agents to mitochondria to achieve enhanced PDT effect and induce favorable immune response [105]. Therefore, nanomaterial will provide a powerful tool to combine the function of PDT in primary cell killing and PDT-induced immunity in secondary cell killing [106].

### 4.3. Experimental Models for PDT-Induced Antitumor Response Study and PDT Evaluation

In order to bring PDT-induced antitumor immunity closer to clinical application, it is crucial to perform preclinical tests to evaluate therapeutic efficacy of PDT based on a platform that can readily mimic human response. As traditional two-dimensional (2D) monolayer static cell culture fails to provide native 3D tissue structure, significant deviation has been noticed in transferring the results from 2D culture to* in vivo* experiments [107–109]. Therefore, animal studies based on tumor-bearing murine models remain tremendously powerful and are considered to be the most common approach for cancer immunology study as well as therapeutic efficacy evaluation of PDT. To minimize rejection by the host immune system so as to obtain desirable tumor formation, human tumor cells are often introduced in immune-suppressed animals lacking key immune players. In order to study important interactions between tumors and immune system as well as their response to specific therapy such as PDT, currently, preclinical research utilized immunocompetent mouse models as a feasible means to study PDT-induced antitumor immunity. Owing to their advantages in providing immune response in a systemic way, majority of studies summarized in our present review are obtained from such type of immunocompetent murine models. Tumor-bearing murine models can be developed in immunocompetent environment through several approaches: syngeneic transplanted tumor model can be achieved by injecting homologous cell lines into immunocompetent mice; spontaneous tumor formation can be induced chemically or by genetic modification in murine model avoiding the host immune rejection; humanized mouse models with immune system can be reconstituted by introducing human dendritic cells, B cells, T cells, or human transgenes that can encode human cytokines [110, 111]. Although these animal studies can provide some predictive guidance in a systematical way, murine models inherently contain non-human host cells, and thus they are still not able to fully recapitulate the physiological or pathological processes in humans. Besides, the development of tumors under immunocompetent conditions often requires long time and high cost accompanying many technique challenges. And intragroup variations in tumor growth in immunocompetent murine models could bring in difficulty in scalability and hamper translation of results from bench to bedside.

Recently, many studies have been devoted to develop* in vitro* 3D culture systems, which represent a more valuable approach for improved preclinical tests, providing integrated analyses of the antitumor efficacy of PDT and PDT-induced immune response* in situ*. These techniques include tumor spheroid formation in scaffolds or hydrogel systems, tumor cell aggregates formation by hanging-drop [112] or bioprinting techniques [113], and tumor tissue growth in microfluidics system [114]. In our previous study, we have developed an* in vitro* 3D breast tumor tissue model by coculturing tumor cells with stromal cells of human origin in the microfluidic system and utilized this model to evaluate the efficiency of PDT under various conditions. By presenting structural, mechanical, and chemical cues, tumor tissues in our microfluidic device are able to mimic heterogeneous tumor microenvironment and emulate physiological conditions [115].

Currently,* in vitro* 3D tissue model is still in its very early developmental stage with focus on the regeneration of single tissue organ. However, more excitingly, this organ-on-a-chip concept can be further extended to body-on-a-chip concept in the near future, which allows for the integration of multiple functional units of human organs to mimic whole body physiology [116–118]. With a controlled medium flow around engineered tissues, it is possible to study the interaction between different organs in a systemic context. In that regard, it highlights the possibilities of using body-on-a-chip technology to study the interaction between tumor tissue and immune system. Given its advantages of high-throughput feature, controllable tumor growth for standardization, and patient-derived cell growth allowing for personalized medicine [119–121],* in vitro* 3D tissue model by tissue engineering technology such as microfluidic technique could be a powerful alternative approach for the study of PDT-induced antitumor immunity.

## 5. Conclusion

PDT is a clinically approved, minimally invasive therapeutic modality for types of tumors. In addition to local tumor destruction and vascular effects of PDT, the mechanisms whereby PDT activates antitumor immunity have been extensively researched. Recognizing the innate potentials of PDT in triggering host immune response, researchers have explored the possibility of various immunotherapeutic strategies in concert with improved efficiency in PDT. However, under what circumstances this PDT-induced immunity can be potentiated to overcome tumor immunosuppression and achieve sufficient antitumor response and to which extent this PDT-induced antitumor immunity will lead to complete tumor rejection still need to be fully elucidated. To answer these questions, a more thorough understanding of the pathways by which tumor cells evade from host immune response and a comprehensive knowledge of dose-dependent effects of different photosensitizers are thus required. While developing combination therapy of PDT and immunosimulating approaches may enhance therapeutic efficacy, intracellular delivery of photosensitizers with the assistance of nanotechnology may favor rational design of PDT regimen. A physiologically relevant tumor tissue model with human derived tumor architecture and heterotypic feature is necessary for the study of tumor mass and immune system interaction as well as PDT-induced antitumor immunity.
